# Median Arcuate Ligament Syndrome (MALS) With a Spontaneous Dissecting Aneurysm of the Celiac Axis

**DOI:** 10.7759/cureus.88766

**Published:** 2025-07-25

**Authors:** Ryan Ward, Devin Naidoo, Shreeja Shikhrakar, Ranjit Chaudhary

**Affiliations:** 1 Radiology, Brown University, Providence, USA; 2 Radiology, Frank H. Netter MD School of Medicine, Quinnipiac University, North Haven, USA; 3 Internal Medicine, St. Vincent's Medical Center, Bridgeport, USA; 4 Radiology, St. Vincent's Medical Center, Bridgeport, USA

**Keywords:** abdominal pain, case report, celiac artery dissection, ct angiography, median arcuate ligament syndrome, vascular compression, visceral aneurysm

## Abstract

We present the fifth documented case, out of a total of four reported in the literature to date, of concurrent median arcuate ligament syndrome (MALS) and celiac artery dissecting aneurysm. A 42-year-old male arrived at the emergency department with acute-onset epigastric and left upper quadrant pain radiating to the left flank, accompanied by nausea without vomiting. Imaging with computed tomography angiography (CTA) confirmed a 1.3 cm dissecting aneurysm of the celiac axis with significant proximal stenosis caused by compression from the median arcuate ligament, consistent with MALS. The diagnosis was based solely on imaging findings; no specific clinical diagnostic criteria were applied. The patient was managed conservatively due to hemodynamic stability, absence of rupture, and stable aneurysmal size on serial imaging. He was discharged with antihypertensives and close outpatient follow-up, with a scheduled vascular surgery consultation in two weeks. We also discuss in this report the previously documented cases of the same or similar occurrences. Across these cases, treatment varied from conservative management to surgical intervention, often influenced by aneurysm stability and rupture risk. Our case adds to the limited literature on this rare presentation and highlights the potential for safe conservative management in select patients. It also underscores the need for diagnostic vigilance and follow-up protocols to better stratify management strategies in MALS-associated vascular complications. These cases emphasize the clinical importance of recognizing the rare coexistence of MALS and celiac artery dissection. Reporting such cases enhances awareness, facilitates early diagnosis, and may inform the development of standardized management approaches.

## Introduction

Median arcuate ligament syndrome (MALS) is an uncommon cause of chronic, recurrent abdominal pain resulting from external compression of the celiac artery by the median arcuate ligament of the diaphragm. Although celiac artery compression is observed in approximately one-third of autopsies, these findings are often incidental and asymptomatic [1,2]. Symptomatic MALS is differentiated by dynamic flow compromise, often during expiration, leading to clinical symptoms such as postprandial abdominal pain, nausea, and weight loss. Hemodynamically significant stenosis, typically demonstrated by imaging criteria such as >70% luminal narrowing or elevated peak systolic velocity on Doppler ultrasound, supports a diagnosis of symptomatic MALS [1,2].

Celiac artery dissecting aneurysm is another rare vascular condition, characterized by a tear in the arterial intima that permits blood to enter and separate the layers of the vessel wall. Recognition of the rare concurrent presentation of MALS and celiac artery dissecting aneurysm is clinically important due to its potential to complicate diagnostic workups and alter management decisions. Additionally, dissecting aneurysms carry a risk of rupture, which may be life-threatening, and the presence of vascular compression can further compromise perfusion or influence the feasibility of surgical or endovascular interventions [3,4]. While the pathophysiological link between MALS and celiac artery dissection is not yet well established, mechanical compression and altered hemodynamics may contribute to aneurysm formation. Chronic compression-induced endothelial injury, post-stenotic dilation, and increased shear stress can all weaken the arterial wall and predispose it to intimal tearing [4-8]. 

This report holds particular relevance for vascular surgeons, radiologists, and emergency physicians who may be the first to evaluate patients with acute abdominal symptoms or vascular abnormalities. While only four previous cases have been reported, they show a spectrum of presentations, primarily middle-aged individuals with acute abdominal pain, with varying management strategies ranging from conservative therapy to surgical intervention [5-8]. Our case adds to this body of literature by expanding understanding of the potential for conservative management in hemodynamically stable patients and reinforces the need for interdisciplinary collaboration in diagnosis and follow-up.

## Case presentation

A 42-year-old male with a past medical history of cervical spine disease, prior hernia repair, and everyday e-cigarette use presented to the ED with abdominal and flank pain. The use of e-cigarettes was noted as a potential vascular risk factor due to emerging evidence linking vaping to endothelial dysfunction and vascular inflammation. On initial presentation, he described an acute onset of severe, constant, tearing pain located in the epigastric and left upper quadrant area, radiating to the left flank, which began the day before and persisted throughout the night. He also reported associated nausea but no vomiting and denied urinary symptoms, fever, chills, cough, or shortness of breath.

Physical examination was significant for an elevated blood pressure of 168/98 mmHg, as well as left upper quadrant and left flank tenderness. Significant laboratory findings included an elevated CRP of 2.80 mg/dL (<1.00 mg/dL), elevated lactic acid of 2.4 mmol/L (0.5-1.9 mmol/L), and slightly elevated alanine aminotransferase (ALT) of 61 U/L (10-49 U/L). These findings were interpreted as markers of systemic inflammatory response and possible visceral hypoperfusion, consistent with a vascular pathology, and they supported a high index of suspicion for ischemic or inflammatory vascular causes. Other results, including a complete blood count, comprehensive metabolic panel, creatinine kinase, troponin I, and urinalysis, were unremarkable.

**Figure 1:**
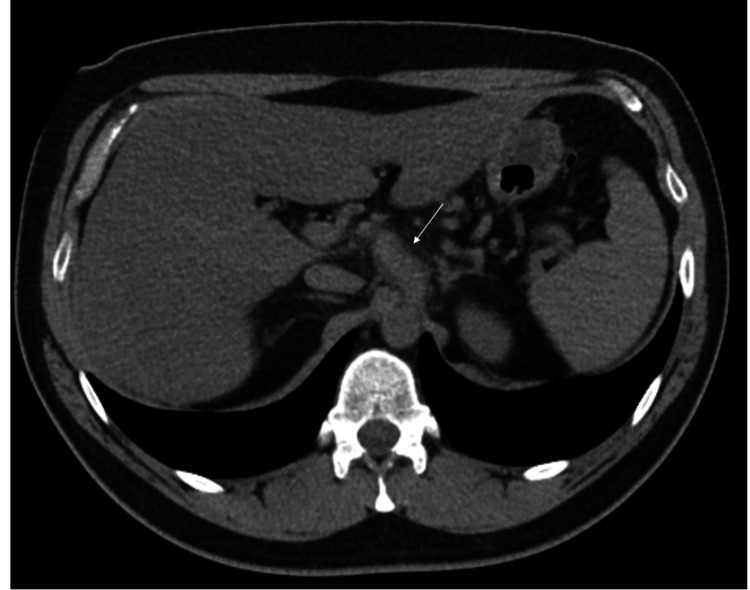
Axial non-contrast CT of the abdomen. Fusiform dilatation of the celiac trunk (white arrow) is evident immediately after its origin from the abdominal aorta. The dilated celiac trunk demonstrates areas of hyperattenuation (+61 HU) and surrounding fat stranding.

Non-contrast CT of the abdomen and pelvis was performed initially, which showed fusiform dilatation of the celiac trunk with surrounding fat stranding (Figure 1). This finding was described as nonspecific, with the explanation that it could signify post-stenotic dilation or fusiform aneurysm formation. A computed tomography angiography (CTA) of the abdomen and pelvis was recommended to differentiate between these possibilities.

**Figure 2:**
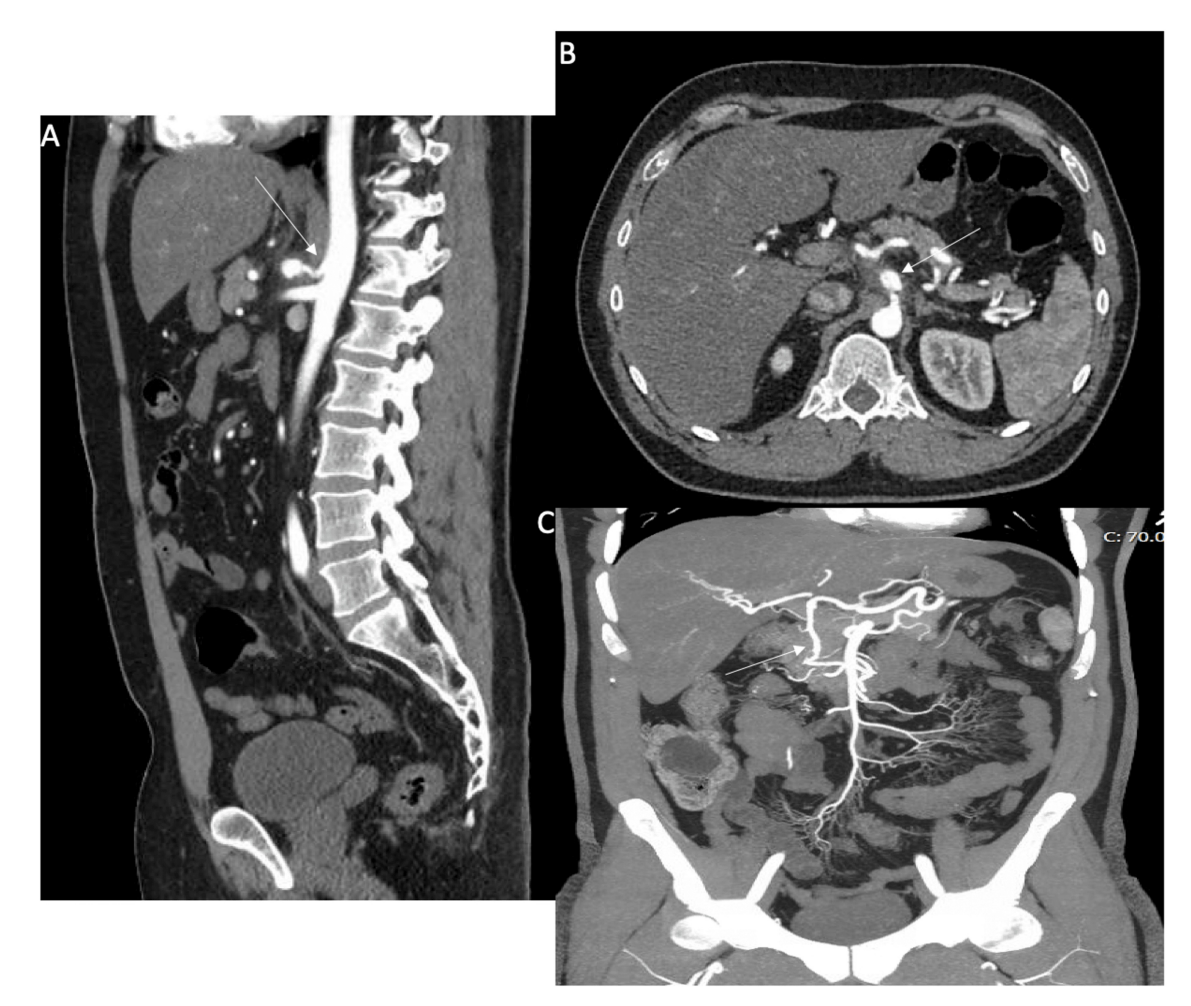
CTA of the abdomen and pelvis. A) Sagittal view shows compression of the celiac artery by the median arcuate ligament (white arrow). B) Axial view shows an enlarged celiac artery distal to the stenosis. A linear filling defect within the aneurysm is suggestive of a dissection flap (white arrow). The hyperdense area seen on the non-contrast study corresponds to a non-enhancing area on CTA surrounding the celiac artery, most likely representing an intramural hematoma. C) Coronal maximum intensity projection (MIP) image demonstrates a collateral vessel (white arrow) between the celiac artery and the superior mesenteric artery. CTA: Computed tomography angiography.

A CTA of the abdomen and pelvis was obtained for further evaluation, which revealed the titular pathology of this case report. The scan showed a 1.3 cm dissecting aneurysm of the celiac axis with severe stenosis proximal to the aneurysm and evidence of compression by the median arcuate ligament of the diaphragm, as the celiac trunk coursed behind it. Additional findings included collateral circulation between the celiac axis and the superior mesenteric artery, suggestive of MALS (Figure 2).

In the ED, the patient received IV ondansetron 4 mg, IV ketorolac 30 mg, IV labetalol 10 mg, oral (PO) metoprolol 25 mg, PO aspirin 81 mg, and PO clopidogrel 150 mg, which resolved both his pain and hypertension during his visit. The use of dual antiplatelet agents was guided by concerns for platelet activation and endothelial injury secondary to the dissection, aiming to mitigate thrombotic propagation within the aneurysmal segment. While not standard for all aneurysm cases, this strategy was considered appropriate given the dissection’s morphology and absence of contraindications.

**Figure 3:**
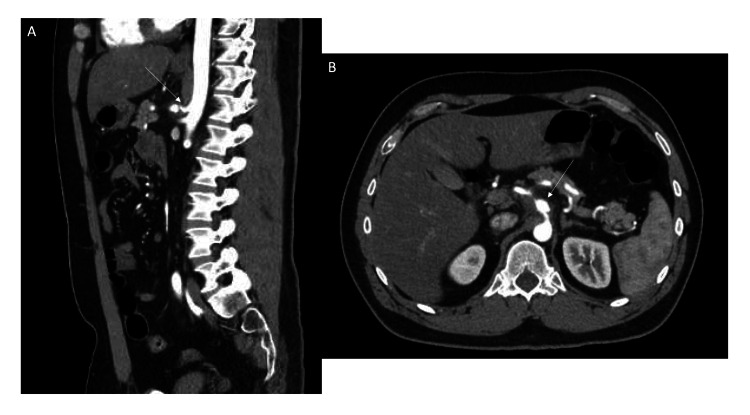
CTA of the abdomen and pelvis at 48 hours. A) Sagittal view shows persistent compression of the celiac artery by the median arcuate ligament (white arrow), with a stable aneurysm of the celiac artery. B) Axial view demonstrates a stable, enlarged celiac artery distal to the stenosis (white arrow), with a surrounding non-enhancing area most likely representing a stable intramural hematoma. CTA: Computed tomography angiography.

Following vascular surgery consultation, inpatient surgery was deferred due to the patient’s hemodynamic stability, lack of rupture, and a preference to observe for spontaneous stabilization. He was admitted to the cardiovascular unit for close monitoring. During hospitalization, he was managed with antihypertensives (hydralazine and escalating doses of carvedilol), analgesia, and continued antiplatelet therapy. Monitoring included frequent vital sign assessments (every 2-4 hours), serial abdominal examinations, and a repeat CTA at 48 hours, which confirmed stability of the dissecting aneurysm, still measuring 1.3 cm (Figure 3).

The patient remained clinically stable with decreasing pain levels. Criteria for discharge on Day 3 included controlled blood pressure (systolic range 126-141 mmHg), resolution of abdominal pain, stable imaging findings, and the ability to continue oral medications. The decision to manage conservatively was supported by the stability of the dissection on serial CTA, the absence of ongoing hemorrhage or organ ischemia, and the patient’s rapid symptomatic improvement with blood pressure control and analgesia. He was discharged with a blood pressure regimen of carvedilol 50 mg, continued dual antiplatelet therapy with aspirin 81 mg and clopidogrel 75 mg, and a scheduled follow-up appointment with vascular surgery in two weeks.

## Discussion

MALS and dissecting aneurysm of the celiac artery are both rare conditions with incompletely understood etiologies. The concurrent presentation of both MALS and dissecting aneurysm of the celiac artery is exceptionally rare, with only four prior cases documented.

In 2012, Watanabe A et al. reported two cases: a 51-year-old male and a 63-year-old female, both of whom presented with acute abdominal pain and were diagnosed with celiac artery stenosis and dissection associated with ruptured pancreaticoduodenal arcade (PDA) aneurysms [8]. Contrast-enhanced CT revealed severe celiac artery compression consistent with MALS and ruptured collateral aneurysms. Both patients underwent transcatheter arterial embolization (TAE) of the PDA aneurysms; however, one patient required additional surgical intervention due to persistent hemorrhage. These cases underscore the importance of prompt recognition and intervention in the setting of vascular rupture.

In 2020, Michell H et al. described a 64-year-old female who presented with epigastric abdominal pain that began during a bowel movement [5]. Contrast-enhanced CT imaging demonstrated hypertrophy of the median arcuate ligament, celiac artery stenosis, post-stenotic aneurysmal dilation, and a dissection flap when compared to prior imaging. As her symptoms had resolved by the time of vascular surgery evaluation, the decision was made to pursue conservative management with daily aspirin and close outpatient follow-up 3-4 weeks after discharge.

In 2021, Li S et al. reported a 54-year-old male with a history of uncontrolled hypertension who presented with three days of abdominal pain and distension and a blood pressure of 180/140 mmHg [7]. CTA revealed a small intimal tear in the celiac artery, a non-ruptured dissecting aneurysm, and significant compression of the celiac axis by the median arcuate ligament. Due to the dissection and imminent risk of rupture, the patient was treated with angiographic celiac trunk stenting, which led to immediate symptom resolution and no complications at one-year follow-up.

In 2022, Saiga A et al. described a case involving a patient of unspecified age and sex who presented with retroperitoneal hemorrhage from a ruptured PDA aneurysm and concurrent celiac artery dissection in the context of MALS [6]. Diagnosis was facilitated by four-dimensional CTA, which showed dynamic compression and hooked narrowing of the celiac artery, more pronounced at end-expiration. The patient was treated with a combination of TAE, celiac stenting, and laparoscopic release of the median arcuate ligament, highlighting the role of multimodal intervention in complex presentations.

These reports, including ours, reveal shared features such as middle-aged patients presenting with acute epigastric pain and celiac artery compression. However, treatment strategies have been individualized in each case. Michell H et al. reported spontaneous symptom resolution and used antiplatelet therapy alone [5]. Watanabe A et al., Li S et al., and Saiga A et al. performed endovascular interventions with either embolization or stenting due to anatomical instability and hemorrhagic risk [6-8]. In the present case, surgical or endovascular intervention was deferred due to stable imaging, controlled symptoms, and the patient’s preference. Regardless, in all reports, patients had favorable outcomes from either conservative treatment or surgical intervention. This underscores the importance of individualized decision-making based on anatomic, clinical, and institutional factors.

While the association between MALS and the development of celiac artery dissecting aneurysm is not definitively established, it is not difficult to hypothesize. It is postulated that chronic mechanical compression of the celiac artery during expiration increases shear stress and induces intimal injury. This may initiate a cascade involving endothelial dysfunction, luminal narrowing, and turbulent flow. Biomechanical models and hemodynamic studies suggest that regions of post-stenotic dilation are prone to wall stress concentrations, which can predispose to both aneurysm formation, via elastin degradation and medial thinning, and to dissection through intimal tear propagation [9,10].

Post-stenotic dilation occurs when elevated upstream pressure and reduced downstream resistance expand the arterial lumen distal to the site of compression. This creates a localized region of altered flow dynamics, potentially leading to mural thrombus formation, weakening of the arterial wall, and separation of intimal and medial layers. Arterial narrowing and stenosis can disrupt blood flow and predispose to dissection and/or aneurysm. Aneurysms secondary to arterial compression and post-stenotic dilation have been described in other compressive syndromes, including thoracic outlet syndrome and popliteal artery entrapment syndrome [9]. Although aneurysm and dissection are distinct processes, they share this hemodynamic vulnerability as a common substrate.

In celiac artery aneurysm, the risk of rupture is high, which can lead to hemorrhage, hemodynamic collapse, and is associated with high mortality [3,4]. Although the true incidence of rupture in dissecting celiac aneurysms remains uncertain, mortality estimates from prior case series range as high as 30% for ruptured lesions [3]. Due to these risks, it is important to utilize imaging that can aid in early diagnosis. Dynamic imaging, particularly four-dimensional CTA, as used by Saiga A et al. [7], offers enhanced visualization of celiac artery compression across respiratory phases. The four-dimensional CTA involved imaging the celiac artery across inspiration and expiration cycles, which revealed dynamic changes in luminal caliber of the celiac artery, including increased compression and characteristic hooked narrowing during end-expiration. This technique improves diagnostic confidence by revealing changes in arterial morphology and perfusion dynamics not visible on static scans. Wider use of dynamic imaging may improve preoperative planning and risk stratification, particularly in ambiguous cases.

This case emphasizes the need for future research initiatives, including prospective registries of visceral artery dissections, longitudinal studies of MALS-associated vascular events, and the development of imaging-based risk scoring systems. Clarifying thresholds for intervention and outcomes of conservative management will be essential to tailoring care strategies for this rare but high-stakes vascular pathology.

## Conclusions

This case highlights a rare but clinically significant concurrence of MALS and celiac artery dissecting aneurysm. Despite the recognized risk of rupture in dissecting aneurysms, conservative management was appropriate in this instance due to the patient’s hemodynamic stability, absence of active hemorrhage, and stability of the aneurysm on repeat imaging. Blood pressure control played a central role in mitigating further hemodynamic stress on the vessel wall, underscoring its importance as a modifiable risk factor in the management of dissecting aneurysms.

Currently, the literature lacks standardized protocols regarding follow-up imaging intervals, blood pressure targets, or surgical thresholds in cases of MALS complicated by arterial dissection. The development of such guidelines, potentially stratified by aneurysm size, growth rate, or symptomatology, would support clinicians in making more evidence-based management decisions.

Future multicenter case registries and prospective studies are essential to more systematically characterize the clinical course, radiologic features, and outcomes of patients with MALS and dissecting aneurysms. These efforts may help establish risk stratification tools, imaging-based scoring systems, and clearer criteria for when to escalate from conservative to surgical intervention.
